# Mecp2-mediated Epigenetic Silencing of miR-137 Contributes to Colorectal Adenoma-Carcinoma Sequence and Tumor Progression via Relieving the Suppression of c-Met

**DOI:** 10.1038/srep44543

**Published:** 2017-03-14

**Authors:** Tao Chen, Shi-Lun Cai, Jian Li, Zhi-Peng Qi, Xu-Quan Li, Le-Chi Ye, Xiao-Feng Xie, Ying-Yong Hou, Li-Qing Yao, Mei-Dong Xu, Ping-Hong Zhou, Jian-Min Xu, Yun-Shi Zhong

**Affiliations:** 1 Endoscopy center, Zhongshan Hospital of Fudan University, Shanghai, 200032, China; 2 Endoscopy Research Institute of Fudan University, Shanghai, 200032, China; 3 Department of Oncological Surgery, the First Affiliated Hospital of Wenzhou Medical University, Wenzhou, 325000, China; 4 Department of Pathology, Zhongshan Hospital of Fudan University, Shanghai, 200032, China; 5 Department of General Surgery, Zhongshan Hospital of Fudan University, Shanghai, 200032, China

## Abstract

The molecular mechanisms underlying colorectal cancer (CRC) development remain elusive. In this study, we examined the miRNA and mRNA expressions in the adenoma-carcinoma sequence (ACS), a critical neoplastic progression in CRC development. We found that miR-137 was down-regulated in all adenoma and carcinoma tissues. Low miR-137 levels were correlated negatively with tumor progression and metastasis. Then we identified the inhibition effect of the miR-137 in CRC development, both in CRC cell lines and mouse models. MiR-137 was shown to control CRC cell proliferation, colony formation, migration and invasion and to control tumor growth and metastasis. We further confirmed the negative association between miR-137 and c-Met expression and thus validated this important oncogene as the target of miR-137 in CRC. In addition, we found a DNA methyl-CpG-binding protein, Mecp2, was up-regulated in ACS tissues via mRNA sequencing. Further experiment showed that miR-137 expression in CRC was subjected to epigenetic regulation mediated by Mecp2. We also confirmed c-Met expression can be up-regulated by silencing of miR-137 and suppressed by coexpression of Mecp2 and miR-137. These findings highlight the critical role of miR-137-c-Met nexus in CRC development and reveal Mecp2-regulated epigenetic silence causes the downregulation of miR-137 in colorectal adenoma and carcinoma.

Colorectal cancer (CRC) is currently one of the most common cancers worldwide and is the third leading cause of cancer-related death^1^. Despite advances and improved understanding in molecular biology, the mechanisms underlying CRC tumorigenesis and progression remain elusive. The colorectal adenoma–carcinoma sequence (ACS) is a gradual progression from the development of colorectal adenomas, to low-grade dysplasia (LGD), high-grade dysplasia (HGD), and eventually, invasive carcinoma^2^,^3^. This stepwise progression is accompanied by successive accumulation of genetic alterations^4^.

MicroRNA (miRNA) is a class of short (18 to 24 nucleotides), non-protein-coding RNA that regulates the translation and degradation of messenger RNA (mRNA) via interacting with its 3′-untranslated region (3′ UTR)^5^. Different patterns of miRNA-expression have been identified in different cancer types^6^. Furthermore, a large body of research showed that miRNA alternations played a key role in the development of various types of cancer. However, little is known about the functional role of miRNA in consecutive colorectal ACS and CRC progressions. In this study, we examined the expression of miR-137 in ACS and explored its role in the regulation of CRC cell function. In addition, miRNA-137-mediated c-Met expression in cells and the underlying mechanism of miRNA-137 alternation in colorectal ACS were also investigated.

## Results

### MiR-137 is associated with ACS and CRC progression

**Figure 1:**
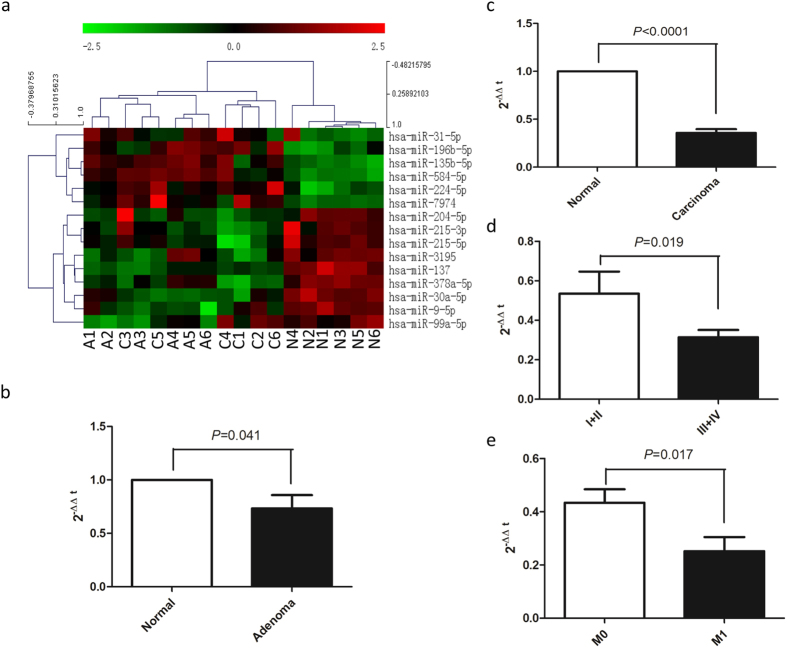
Cluster analysis of aberrant miRNA expression in colorectal ACS according to a small RNA sequencing and qQRT-PCR validation of miR-137 expressions in human tissues. (**a**) dendrogram generated by cluster analysis showing the differential expression of miRNAs in ACS (>2 fold changes). (**b**) miR-137 expression was significantly decreased in colorectal adenoma. (**c**) miR-137 expression was significantly decreased in CRC tissues. (**d**) decreased miR-137 expression was correlated with CRC TNM stage. (**e**) decreased miR-137 expression was correlated with CRC metastasis. N, normal tissue; A, adenoma; C, carcinoma.

A small RNA sequencing analysis of 18 colorectal ACS tissues from 6 patients was conducted to study the effect of miRNA profile in regulating human colorectal ACS and CRC progression. We identified 15 miRNAs that had at least 2-folds higher expression levels compared with other groups (Fig. 1a). MiR-137 was found to be consistently down-regulated in 6 pairs of adenoma and carcinoma tissues. QRT-PCR analysis of miR-137 expression in 30 colorectal adenoma tissues and in 70 CRCs showed miRNA-137 was not only differentially expressed in colorectal adenoma (Fig. 1b; P = 0.041), but also significantly reduced in tumor tissues (Fig. 1c; P < 0.001). When the clinicopathological implication of miR-137 was analyzed in CRC patients it is found that low miR-137 levels were negatively correlated to tumor TNM stage (Fig. 1d; P = 0.019) and metastasis (Fig. 1e; P = 0.017).

### Over expression of miR-137 inhibits CRC cell proliferation, colony formation, migration, and invasion *in vitro*

**Figure 2:**
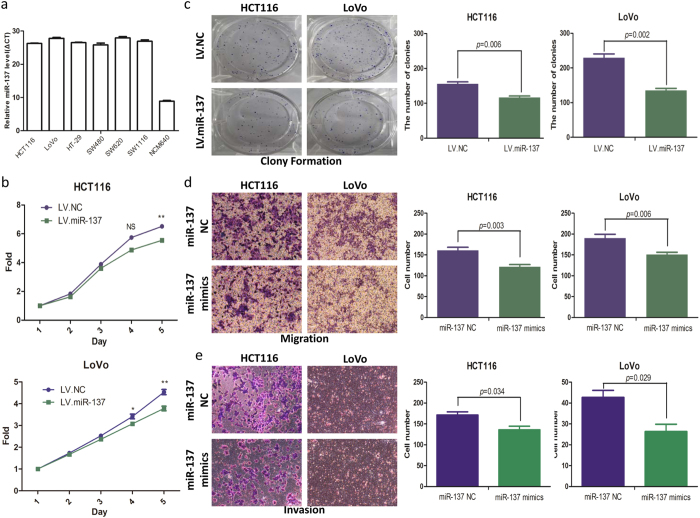
Effect of miR-137 on proliferation, colony formation, migration, and invasion of HCT116 and LoVo cells. (**a**) miR-137 levels in 6 CRC cell lines and the colon mucosa cell line NCM640. (**b**) overexpressed miR-137 had significant effect on decreasing proliferation rate of both cell lines. (**c**) The number of clones of HCT116 and LoVo with overexpressed miR-137 was fewer than that of control cells. (**d**) Representative fields of migration cells on the membrane were on the left (magnification of 200×). Average migration cell number per field was on the right. (**e**) Representative fields of invasion cells on the membrane were on the left (magnification of 200×). Average migration cell number per field was on the right.

The biological role of miR-137 was examined *in vitro* by functional assays. Expression of miR-137 in 6 CRC cell lines and colon mucosa cell line NCM640 was shown in Fig. 2a. A significant decrease in cell proliferation was observed in both miR-137 lentivirus (LV.miR-137)-infected HCT116 and LoVo cells comparing with the negative control (LV.NC) (Fig. 2b). In both cell lines, colony formation ability was inhibited by the overexpression of miR-137 (Fig. 2c). MiR-137 mimics were transfected into HCT116 and LoVo cell to transiently increase the miR-137 expression. The results from cell migration and invasion assays showed that the overexpression of miR-137 significantly inhibited HCT116 and LoVo cell migration and invasion via cell migration and invasion assays (Fig. 2d,e). Taken together, we have shown the tumor suppressor role of miR-137 in CRC development.

### Overexpression of miR-137 inhibits colorectal tumor growth and hepatic metastasis *in vivo*

To further investigate the *in vivo* effect of miR-137 on tumor formation and metastasis, HCT116 cells, which were stably transfected with either LV.miR-137 or LV.NC, were subcutaneously and intraspleenically injected into nude mice.

**Figure 3:**
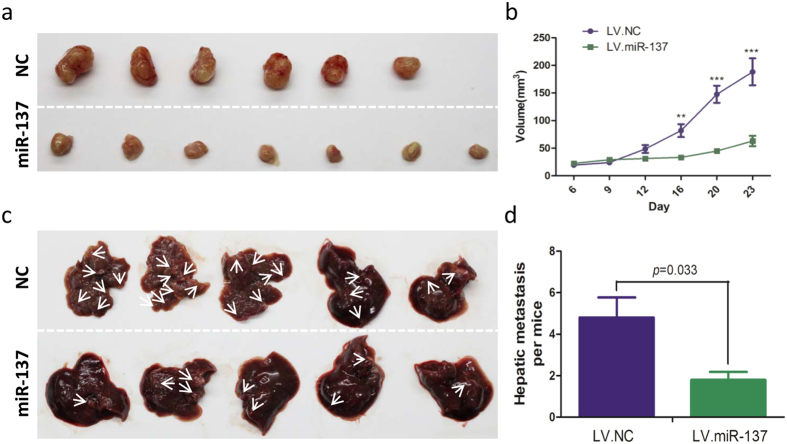
MiR-137 controls the tumor growth of HCT116 xenografts and hepatic metastasis *in vivo*. (**a,b**) The tumors were much bigger in control group than that in overexpressed miR-137 group. (**c,d**) The injection of HCT116 cells with overexpressed miR-137 into spleen led to the formation of more metastase nodes in the liver. **P < 0.01, ***P < 0.001.

At 4 weeks post-injection, the average tumor size was found to be significant smaller in rats injected with cells that overexpressed miR-137 compared with the control group (Fig. 3a,b). Similarly, less hepatic metastasis nodes were found in rats with cells that overexpressed miR-137 (Fig. 3c,d). In coordination with *in vivo* study, overexpression of miR-137 was found to inhibit colorectal tumor progression and hepatic metastasis as well.

### MiR-137 alterations affect ACS and tumor progression by down regulating c-Met expression

**Figure 4:**
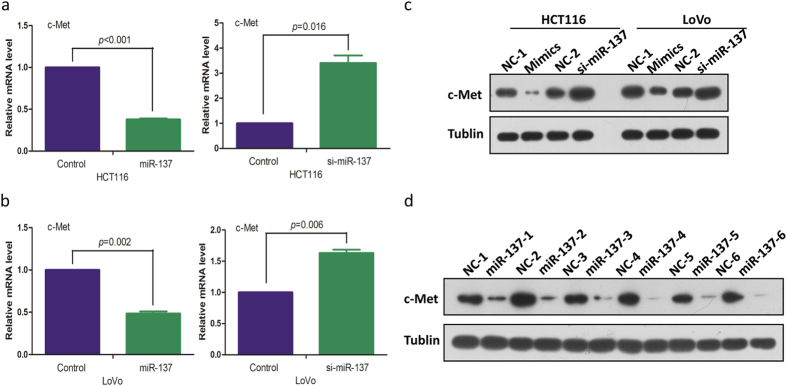
C-Met is one of the miR-137 targets and is negatively regulated by miR-137. (**a**) c-Met expression in HCT116 cells after transfection with miR-31 mimics (left) or anti-miR-31 siRNA (right) detected by qQRT-PCR. (**b**) c-Met expression in LoVo cells after transfection with miR-31 mimics (left) or anti-miR-31 siRNA (right) detected by qQRT-PCR. (**c**) c-Met expression in HCT116 and LoVo cells after transfection with miR-137 mimics or anti-miR-137 siRNA detected by Western blot. (**d**) c-Met expression was negatively correlated with miR-137 in 6 pairs of xenografts.

Both Target Scan and Pictar systems were used to identify putative gene targets of miR-137. C-Met was selected for further analysis because of its involvement in various malignances and was predicted to be a target of miR-137 in melanoma^7^. In vitro and *in vivo* assays were conducted to determine if miR-137 could regulate c-Met expression in CRC. HCT116 and LoVo cells were transfected with miR-137 or control mimics respectively. Forced expression of miR-137 was associated with reduce c-Met mRNA levels in both cell lines (Fig. 4a,b). Anti-miR-137, and anti-miRNA control were then transfected into the above 2 cell lines. Lower expression of miR-137 increased c-Met mRNA levels in both cell lines (Fig. 4a,b). Cell lysate were analyzed with Western blot to determine the protein levels of c-Met in the aforementioned treated cells and xenograft tumors. We found that the expression of both c-Met was negatively correlated with miR-137 (Fig. 4c,d).

### Mecp2-regulated epigenetic silencing of miR-137 contributes to colorectal ACS and tumor progression by relieving the suppression of c-Met

**Figure 5:**
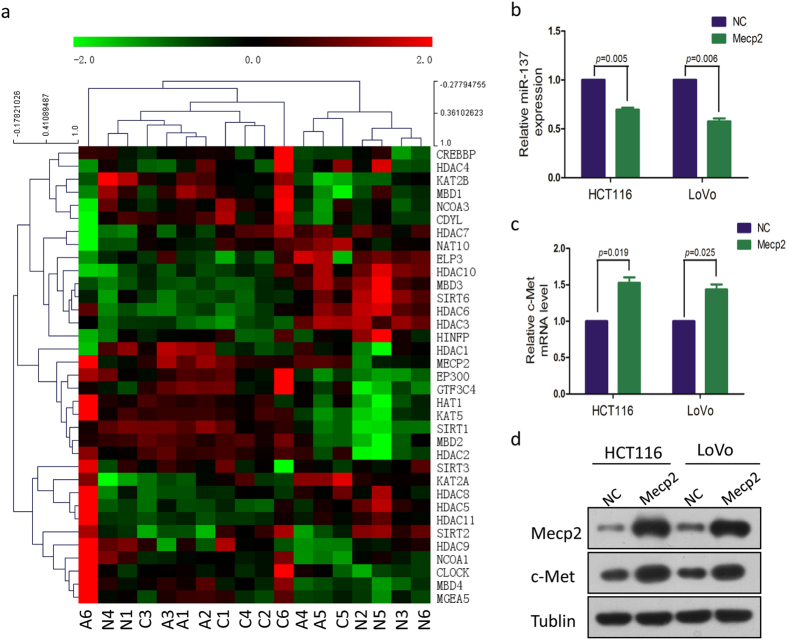
Cluster analysis of aberrant mRNA expression in colorectal ACS according to a mRNA sequencing and validation of Mecp2 regulating miR-137 expressions in CRC cell lines. (**a**) dendrogram generated by cluster analysis showing the differential expression of mRNAs in ACS (>2 fold changes). (**b**) miR-137 expression in both HCT116 and LoVo cells with overexpressed Mecp2. (**c**) c-Met mRNA expression in both HCT116 and LoVo cells with overexpressed Mecp2. (**d**) c-Met expression in both HCT116 and LoVo cells with overexpressed Mecp2 Western blot. N, normal tissue; A, adenoma; C, carcinoma.

**Figure 6:**
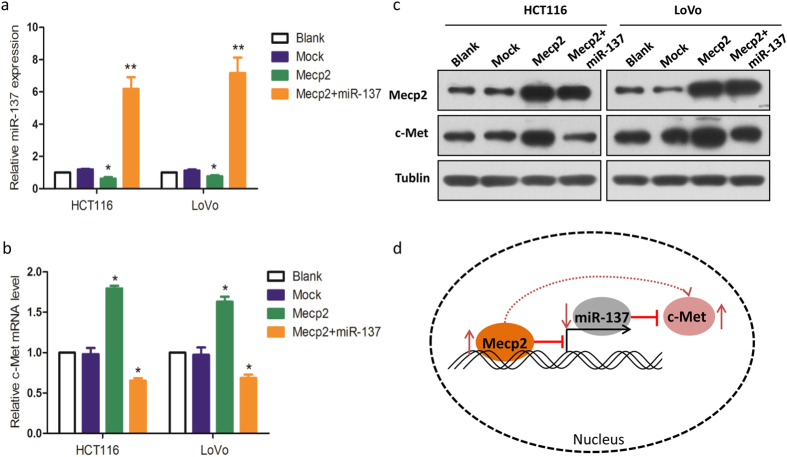
Overexpressed Mecp2 relieved the suppression of c-Met by miR-137. (**a**) miR-137 expressions in HCT116 and LoVo cells with transfected Mecp2 overexpression plasmid, or transfected both Mecp2 overexpression plasmid and miR-137 mimics. (**b**) c-Met mRNA expressions in HCT116 and LoVo cells with transfected Mecp2 overexpression plasmid, or transfected both Mecp2 overexpression plasmid and miR-137 mimics. (**c**) Mecp2 and c-Met protein expressions in HCT116 and LoVo cells with transfected Mecp2 overexpression plasmid, or transfected both Mecp2 overexpression plasmid and miR-137 mimics. (**d**) schematic of the entire signaling pathway described, including the interaction of Mecp2, miR-137, and c-Met. The epigenetic silencing of miR-137 caused by the upregulation of Mecp2 induces c-Met expression and contributes CRC development. In addition, Mecp2 was suggested to directly bind to c-Met and to upregualate c-Met expression.

MiR-137 is constitutively expressed in colonic epithelium and epigenetic silencing of miR-137 is an early event in colorectal carcinogenesis^8^. To study the epigenetic profile and its role in regulating human ACS, a transcriptome analysis in 18 colorectal ACS tissues collected from the same 6 patients was performed. Thirty five epigenetic genes with a fold-change of >2 in the between group expression levels were collected. Mecp2 was found to be consistently up-regulated in ACS tissues, compared to the normal tissues (Fig. 5a). Mecp2 is a DNA methyl-CpG-binding protein and was reported to epigenetically regulate miR-137 in adult neural stem cells^9^. In the current study, a ChIP assay was performed and we confirmed that Mecp2 could directly bind to the promoter of miR-137 (Fig. S1). In addition, we overexpressed Mecp2 in HCT116 and LoVo cells and found that miR-137 expression was significantly decreased (Fig. 5b). Furthermore, the expression of c-Met, the target of miR-137, was significantly increased because of inhibition of miR-137 (Fig. 5c). The results were consistent with the aforementioned Western blot assay data (Fig. 5d). To further confirm the deduction, Mecp2 plasmid and miR-137 mimics were transfected into HCT116 and LoVo cells. The expressions of c-Met were suppressed again when the miR-137 expression was recovered (Fig. 6a), (Fig. 6b,c). These results suggest a regulatory cascade from MeCP2, miR-137, to c-Met exists (Fig. 6d).

## Discussion

The tumor miRNA profile may resemble that of their antecedent stem cells and thus reflect development lineage^10^. A study conducted by Lu *et al*.^6^ examined the expression of miRNAs across normal tissues, primary tumors as well as cell lines and indicted that tumors display a miRNA expression profile reminiscent of that in the tissues from which they were derived. Therefore, the miRNA may play a role in determination and/or maintenance of lineage during tumor development. In this sense, we collected 18 ACS samples from 6 patients and employed a small RNA sequencing to examine the differential expressions of miRNAs in them. To control the tumor heterogeneity, 3 consecutive ACS samples were collected from one patient. We found miR-137, a key molecular in cross talk between microRNAs and epigenetic regulation in the carcinogenesis^9^, was significantly down-regulated in colorectal adenomas and carcinomas, comparing to normal tissues. It has been reported that miR-137 functioned as a tumor suppressor in cancer types such as head-neck cancer and gastric cancer^11^,^12^. However, its effect in colorectal ACS remains obscure. In this study, we focus our investigation on the role of miRNAs in colorectal ACS via small RNA sequencing analysis and found low expression of miR-137 may be involved in. The miR-137 whose expression was down-regulated in colorectal adenomas and carcinomas was further validated. In addition, the miR-137 level was significantly correlated with CRC progression. To identify the role of the miR-137 in CRC development, functional assays were performed and aberration of miR-137 expression was shown to alter cell proliferation, colony formation, migration, and invasion in CRC cell lines as well as tumor growth and liver metastasis in mouse models.

MiRNAs functions as post-transcriptional regulation of gene expression by complementary base pairing with the 3′UTR of target mRNAs, causing their degradation or suppressing mRNA translation^13^. Previous studies found that miR-137 is negatively correlated with mRNAs^7^,^8^. We selected c-Met as a candidate target of miR-137 for its role in the progression of a wide variety of human malignancies^14^. The findings strongly suggested miR-137 could directly target c-Met and regulate c-Met expression *in vitro* and *in vivo*. Furthermore, the results obtained from qRT-PCR and Western blot analysis confirmed both mRNA and protein of c-met are negatively associated with miR-137 expression. Takeuchi *et al*.^15^ found the mRNA expression of c-Met in CRC specimens was significantly higher than that in colorectal normal mucosa tissues. Additionally, colorectal adenomas showed different levels of c-Met mRNA expression apart from all colorectal carcinomas^15^. The c-Met pathway was implicated to be related to colorectal normal mucosa, colorectal adenoma, and colorectal carcinoma progression^15^.

Aberrant epigenetic regulation of miR-137 promoter, such as DNA hypermethylation, may represent a key mechanism for miR-137 down-regulation in several human cancers^16^. Epigenetic silencing through promoter methylation of miR-137 is an early event in colorectal adenomas and the following carcinogenesis^8^. Results from silico analysis have found several epigenetic genes altered in ACS. In this study, we focused on Mecp2 which was reported to epigenetically regulate specific miRNAs in adult neural stem cells^9^. Our results suggested miR-137 expression in CRC was subjected to epigenetic regulation mediated by Mecp2. We also found c-Met expression could be up-regulated by silencing of miR-137 and suppressed by coexpression of Mecp2 and miR-137. Identification of these gene alterations has provided new sights into the molecular process of developmental course from colorectal adenoma to carcinoma. In addition, in the study by Plummer *et al*.^17^, they indicated that Mecp2 could directly bind to the c-Met promoter and activate its transcription. In the current study, our results suggested Mecp2 could upregulate c-Met expression by silencing miR-137 expression (Fig. 6d). Thus, the relationship between Mecp2 and c-Met and the regulation mechanism needed to be further investigated.

Taken together, our results identified a new regulatory network of the miR-137/c-Met nexus, which is not reported in ACS but functions as an oncogenic miRNA in CRC development. More than that, an important funding regarding the significant roles that crosstalk between miRNAs and epigenetics might played in ACS and tumor progression was illustrated. Further understanding in pathogenic significance for epigenetic regulation of miRNAs and its functional targets will inspire novel thinking in diagnosis and treatment of CRC.

## Materials and Methods

### Human tissue samples

Human tissue samples were collected from 6 patients with both colorectal adenoma and carcinoma for small RNA and mRNA sequencing, 30 patients with colorectal adenomas and 70 patients with CRCs. All tissue samples were snap frozen in liquid nitrogen at the time of collection and then stored at -80 °C for later use. The diagnosis of colorectal adenoma and carcinoma was confirmed through pathological examination. All clinicopahtological data were collected (Tables S1 and S2). This study was conducted under the principles of the World Medical Association Helsinki agreement. Ethical approval was obtained from the Ethics Committee of Zhongshan Hospital, Fudan University. Written informed consents were obtained for experimentation with human subjects prior to recruiting participants and conducting procedures.

### Quantitative real-time PCR Assay and RNA sequencing

Total RNA was isolated with TRIzol reagent (Invitrogen) according to the manufacturer’s instructions. Quantitative TaqMan real-time PCR assays for miR-137 (5′-CAAATTCGTGAAGCGTTCCATAT-3′), c-Met (forward, 5′-AGTCATAGGAAGAGGGCATT-3′, and reverse, 5′-CTTCACTTCGCAGGCAGA-3′), Mecp2 (forward, 5′-AAGTGGAGTTGATTGCGTAC-3′, and reverse, 5′-TGGGCTTCTTAGGTGGTT-3′), and GAPDH (forward, 5′-TGACTTCAACAGCGACACCCA-3′, and reverse, 5′-CACCCTGTTGCTGTAGCCAAA-3′) were conducted. All reactions, including no-template controls, were run in triplicates. After the reactions were completed, CT (cycles to threshold) values were determined using fixed threshold settings. Data were analyzed based on the 2^−ΔΔCT^ method. Library preparation for both small RNA and mRNA sequencing was performed according to the manufacturer’s instructions. Briefly, total RNA was isolated from colorectal adenoma and carcinoma tissues with TRIzol reagent (Invitrogen) and analyzed on a 2100-Bioanalyzer (Agilent Technologies) to determine quantity. Highquality total RNA (1 ug) was used as the starting material. Sequencing was performed using HiSeq2500 (Illumina Inc., San Diego, CA) at Genergy Biotechnology (Shanghai) Co., Ltd.

### Cell culture and Proliferation assay

HCT116 and LoVo human CRC cells were obtained from Shanghai Institutes for Biological Sciences, Chinese Academy of Sciences. HCT116 cells were cultured in McCoy’s 5 A medium supplemented with 10% fetal bovine serum (FBS). LoVo cells were cultured in RPMI1640 medium supplemented with 10% FBS. All cells were cultured in a humidified incubator at 37 °C with 5% CO_2_ and plated on 96-well plates at 1 × 10^3^ cells/well. The proliferation of cancer cells 5 days after treatment were measured by the 3-(4,5-dimethylthiazol-2-yl)-2,5-diphenyl tetrazolium bromide (MTT) assay.

### Colony Formation Assay

Colony Formation Assay was carried out according to standard procedures. Cells were mixed with culture medium containing 0.6% agar to achieve a final agar concentration of 0.4%. 1 mL of the cell suspension was immediately plated onto 6-well plates that were coated with 0.6% agar in tissue culture media at 1 mL/well. Cell colonies were counted in triplicates at day 15.

### Cell migration and invasion assays

24-well transwell plate (8-mm pore size, Corning) was used to measure the migratory and invasive ability of each cell line. For migration assay, 5 × 10^4^ cells were plated onto the top chamber lined with a non-coated membrane. For invasion assay, chamber inserts were coated with 200 mg/ml of Matrigel, which were allowed to dry overnight under sterile conditions. 1 × 10^5^ cells were plated onto the top chamber. Both assays were carried out according to standard procedures.

### Overexpression and knockdown of miR-137

Cells were seeded onto 6-well plates at a density of 2 × 10^5^ per well and were given at least 16 hours to allow cell attachment to the well surface before consucting experiments. siRNA or negative control was transfected with lipofectamine 2000 (Invitrogen). Stable cell lines were established by lentiviral infection (LV). LV-miR-137, si-miR-137, and miR-137 mimics were purchased from GeneChem, Shanghai China.

### Protein extraction and western blotting

Cellular proteins were extracted and separated in SDS-PAGE gels, and Western blot analyses were performed according to standard procedures. The membranes were immunoblotted with the following antibodies: anti-c-Met (1:500, Abcam), anti-Mecp2 (1:500, Abcam), and anti-Tublin (1:1000, Abcam). As a loading control, GAPDH detection was performed with primary antibody (1:5000, Abcam).

### Chromatin immunoprecipitation

HCT116 cells were grown on 3–6 confluent 6 cm cell culture plates and were fixed by 1% formaldehyde to culture medium for 10 min at room temperature. Cell lysates were pelleted by centrifugation at 3,000 rpm for 5 min, resuspended again in 1 ml of cold cell lysis buffer for 5 min on ice, and then repelleted to collect nuclei. ChIP was performed as described previously^9^. The Pierce Agarose ChIP Kit (Thermo) was used. For qPCR followed by ChIP, the Premix Taq™ Kit (Takara) was used. Primers spanning 200–250 bp of miR-137 promoter were used for the ChIP qPCR (Supplementary Table S3). Primer sequences spaced at 250 bp intervals spanning 1.0 kb from upstream of miR-137 open reading frame (ORF) were designed using Primer 5.0 software (Premier).

### Immunodeficient mouse model

All animal experiments were approved by the animal care and use committee of Fudan University and performed in accordance with the guidelines of the National Institutes. HCT116 cells infected with LV-miR-137 or LV-NC were harvested and injected into female nude (nu/nu) mice (5 × 10^6^ viable tumor cells/mouse for the subcutaneous xenograft tumor model and 1 × 10^6^ viable tumor cells/mouse for the intraspleenic liver metastasis model). The animals were equally divided into a treatment group and a control group (8 mice per group for the subcutaneous xenograft tumor model and 5 mice per group for the intraspleenic liver metastasis model). Based on length and width measurements, xenograft tumor volume (*V*) was calculated by *V* = (length × width2)/2 every 4 days. During this period, in the study of subcutaneous xenograft tumor model, there were 2 mice dead in the control group with and 1 mouse dead in the treatment group. Subcutaneous tumor volume and hepatic metastasis nodes were calculated after the mice were sacrificed 28 days post-injection.

### Statistical analysis

All experiments were performed in triplicates and all data were expressed as mean ± SD. Differences between groups were estimated by Student’s t-test or Fisher’s exact test at a *p*-value threshold of 0.05. All statistical analyses were performed with SPSS software version 17.0 (SPSS, Chicago, IL, USA).

## Additional Information

**How to cite this article**: Chen, T. *et al*. Mecp2-mediated Epigenetic Silencing of miR-137 Contributes to Colorectal Adenoma-Carcinoma Sequence and Tumor Progression via Relieving the Suppression of c-Met. *Sci. Rep.* **7**, 44543; doi: 10.1038/srep44543 (2017).

**Publisher's note:** Springer Nature remains neutral with regard to jurisdictional claims in published maps and institutional affiliations.

## Supplementary Material

Supplementary Information
