# Supplementary material for: Mecp2-mediated Epigenetic Silencing of miR-137 Contributes to Colorectal Adenoma-Carcinoma Sequence and Tumor Progression via Relieving the Suppression of c-Met
Source: Sci Rep. 2017 Mar 14;7:44543. doi: 10.1038/srep44543 (PMC5349564; doi:10.1038/srep44543)

Supplementary Materials

Mecp2-mediated Epigenetic Silencing of miR-137 Contributes to Colorectal Adenoma-Carcinoma Sequence and Tumor Progression via Relieving the Suppression of c-Met

Tao Chen, Shi-Lun Cai, Jian Li, Zhi-Peng Qi, Xu-Quan Li, Le-Chi Ye, Xiao-Feng Xie, Ying-Yong Hou, Li-Qing Yao, Mei-Dong Xu, Ping-Hong Zhou, Jian-Min Xu, Yun-Shi Zhong

Supplementary Table 1 Clinicopathological characteristics of 6 patients with colorectal adenomas and carcinomas for small RNA and transtriptome sequencings.

Case	Gender	Age	Tumor Location(A/C)	Adenoma Grade	Carcimoa Differentiation	Carcinoma Stage
1	Male	64	Colon R/Colon R	Low	Level II	T3N0M0
2	Female	57	Colon R/Colon R	Low	Level II	T3N0M0
3	Male	50	Rectum/Rectum	Low	Level III	T3N0M0
4	Male	47	Colon L/Rectum	Low	Level II-III	T4N0M0
5	Male	52	Colon L/Rectum	Low	Level II	T3N0M0
6	Female	57	Colon L/Colon R	Middle	Level II	T3N0M0

Supplementary Table 2 Clinicopathological characteristics of 70 patients with CRC for validation.

Total (n)	70
Gender (n)	
Male	45
Female	25
Age (year), mean±SEM	60.81±10.97)
Tumor Location (n)	
Right colon	23
Left colon	16
Rectum	31
Differentiation (n)	
Poorly	20
Level II	32
Level II-III	18
Tumor volume (cm^3), mean±SEM	9.39±8.39
T stage (n)	
2	4
3	16
4	50
N stage (n)	
0	30
1	18
2	22
M stage (n)	
0	41
1	29

Supplementary Table 3 The primers used in ChIP assay

Primer 1	forward	TAAAATCACGATCAACAATTAG
	reverse	CAGTGAGGAGATGAGAAGAGT
Primer 2	forward	TCTCCTCACTGCAATCATAC
	reverse	CAAGATCAAACTCTGCCATAT
Primer 3	forward	AGAAAAGAAAATGGGGATTG
	reverse	AAACATGCTAATGGTGGATT
Primer 4	forward	CATTGGGAAGTCATGAATAA
	reverse	TGATCTACACCATCTGTCGC

Supplementary Figure 1 MeCP2 directly binds to the miR-137 promoter. (a) schematic of the 5' promoter region of miR-137. Horizontal lines indicate primers used in ChIP assays (gray); TSS, miR-137 transcriptional start site. (b) anti-Mecp2 antibody directly pulls down the miR-137 promoter sequence using primers 1, 2 and 4 (red bars).

Figure S1

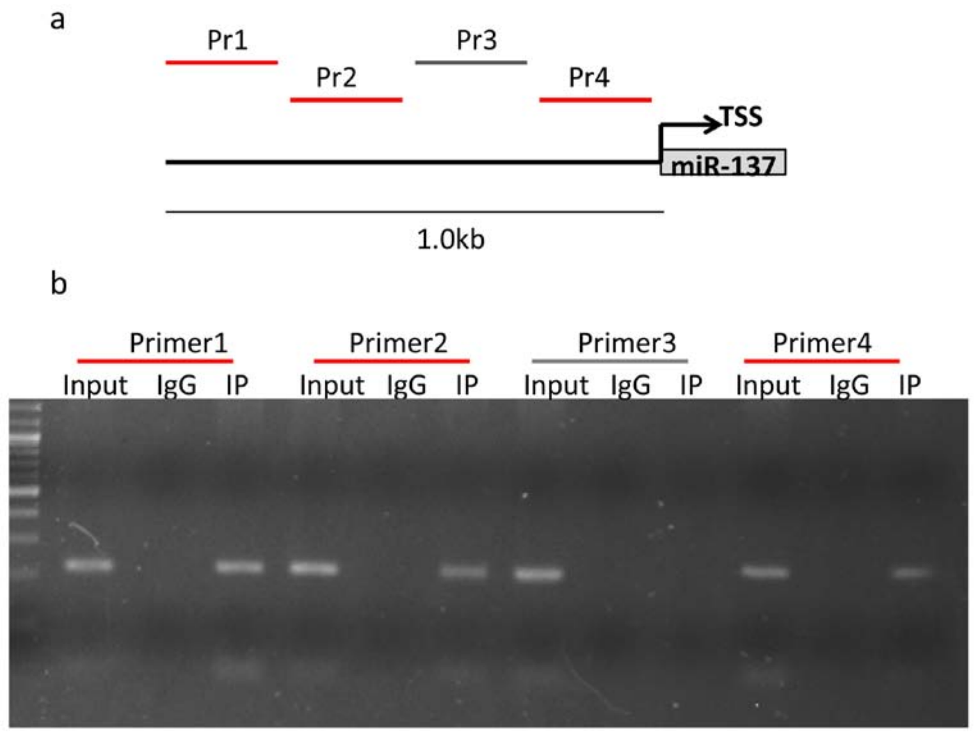

Supplementary Figure 2 The parent unmodified Western blot images without cropping. (4c) Western blot analysis of c-Met expression in HCT116 and LoVo cells after transfection with miR-137 mimics or anti-miR-137 siRNA. (4d) Western blot analysis of the correlation of c-Met protein levels with overexpressed miR-137 in 6 pairs of xenografts. (5d) Western blot analysis of c-Met expression in both HCT116 and LoVo cells with overexpressed Mecp2. (6c) Western blotting analysis of Mecp2 and c-Met protein expressions in HCT116 and LoVo cells with transfected Mecp2 overexpression plasmid, or transfected both Mecp2 overexpression plasmid and miR-137 mimics.

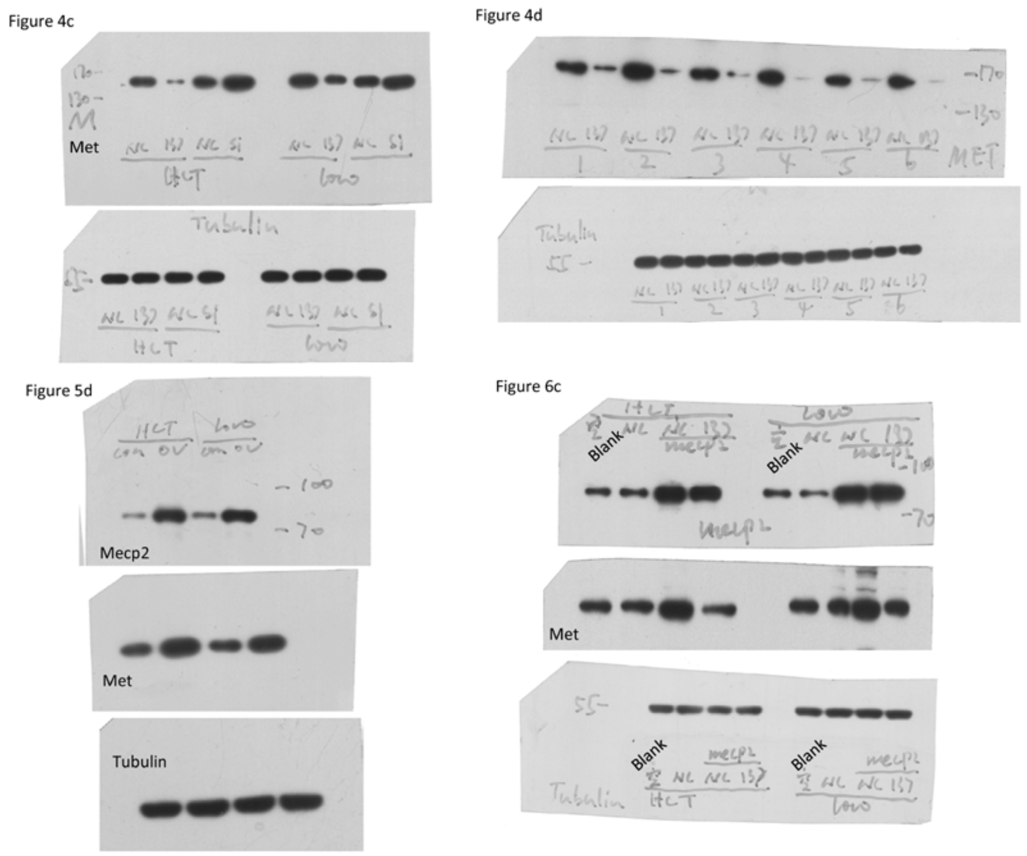

Supplementary Figure 3 Gel showed anti-Mecp2 antibody directly pulls down the miR-137 promoter sequence using primers 1, 2 and 4.

Figure S1

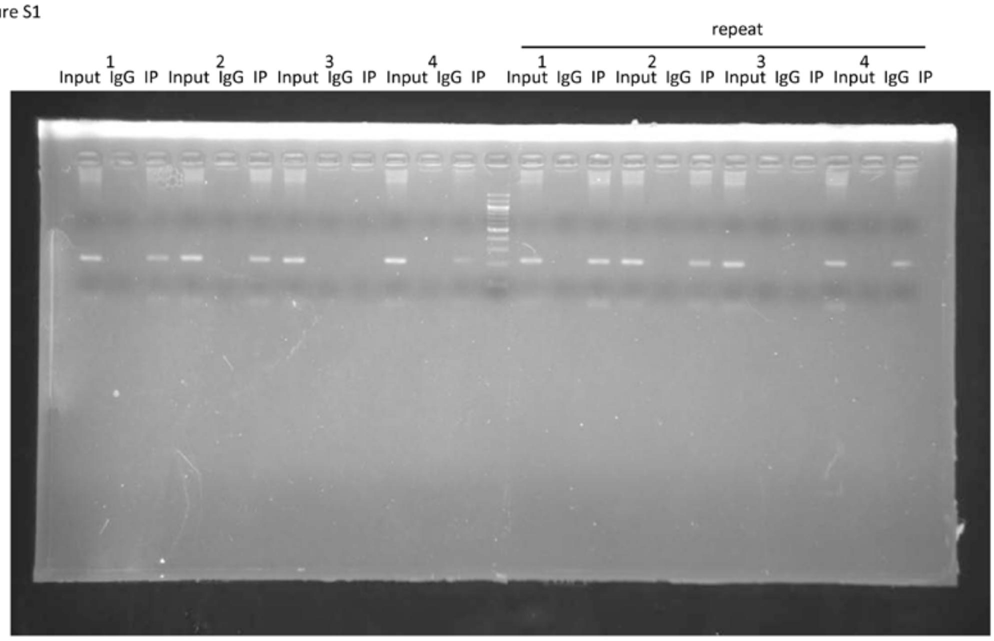

Supplement: Supplementary Information [file srep44543-s1.pdf]
